# Arsenic Species in Edible Seaweeds Using *In Vitro* Biomimetic Digestion Determined by High-Performance Liquid Chromatography Inductively Coupled Plasma Mass Spectrometry 

**DOI:** 10.1155/2014/436347

**Published:** 2014-03-09

**Authors:** Yan-Fang Zhao, Ji-Fa Wu, De-Rong Shang, Jin-Song Ning, Hai-Yan Ding, Yu-Xiu Zhai

**Affiliations:** ^1^Yellow Sea Fishery Research Institute, Chinese Academy of Fishery Sciences, Nanjing Road 106, Qingdao 266071, China; ^2^Ocean and Fishery Bureau of Huangdao District of Qingdao City, Qingdao 266400, China

## Abstract

Arsenite [As (III)], arsenate [As (V)], methylarsonate (MMA), and dimethylarsinate (DMA) in five edible seaweeds (the brown algae *Laminaria japonica*, red algae *Porphyra yezoensis*, brown algae *Undaria pinnatifida*, brown algae *Hizikia fusiformis*, and green algae *Enteromorpha prolifera*) were analyzed using *in vitro* digestion method determined by high-performance liquid chromatography inductively coupled plasma mass spectrometry. The results showed that DMA was found in the water extracts of all samples; As (III) were detected in *L. japonica* and *U. pinnatifida* and about 23.0 and 0.15 mg/kg of As (V) were found in *H. fusiformis* and *E. prolifera* respectively. However, after the gastrointestinal digestion, As (V) was not detected in any of the five seaweeds. About 0.19 and 1.47 mg/kg of As (III) was detected in the gastric extracts of *L. japonica* and *H. fusiformis*, respectively, and about 0.31 and 0.10 mg/kg of As (III) were extracted from the intestinal extracts of *Porphyra yezoensis* and *U. pinnatifida*, respectively. The present results successfully reveal the differences of As species and levels in the water and biomimetic extracts of five edible seaweeds. The risk assessment of the inorganic arsenic in the five edible seaweeds based on present data showed almost no hazards to human health.

## 1. Introduction

Seaweed is known to contain high concentrations of arsenic in comparison with terrestrial plants because marine plants possess the ability to concentrate the arsenic derived from water. Arsenic exists in different chemical forms, which are either “free” inorganic arsenic species, such as As (III) and As (V), or organic arsenic species, such as arsenobetaine (AsB) and arsenosugars [1]. As (III) is generally recognized as being more toxic than As (V), and the toxicity of inorganic arsenic is about 100 times greater than that of monomethylarsonic acid (MMA) or dimethylarsinic acid (DMA). The 50% lethal dose (LD_50_) values of the different forms of arsenic in mg/kg are as follows: As (III) (14) > As (V) (20) > MMA (V) (700–1800) > DMA (V) (700–2600) > AsC (>6500) > AsB (>10000) [2]. Based on these data, we believe that the levels of As (III), As (V), MMA, and DMA in edible seaweeds should be researched in greater detail because of the potential risks to human health. The arsenosugars which comprise the major arsenic species in seaweed are considered nontoxic [2]. In addition, arsenosugars are difficult to obtain because of their chemical synthesis, or complex procedures of isolation from algae in a pure form. So there have no arsenosugars standard materials commercially available by now. Based on these reasons, arsenosugars are not quantified and discussed in present research.

Asian cultures have traditionally employed seaweeds (macroalgae) as a natural source of food and medicines, and Japan, Korea, China, Vietnam, Indonesia, and Taiwan are by far the largest consumers of seaweeds in the world. Marine algae are rich in a variety of different nutrients, including iodine, vitamins, amino acids, dihomo-*γ*-linolenic acid, essential polysaccharides, dietary fiber, and trace elements. Marine algae have also been reported to possess antioxidant and antitumor activities, as well as being linked to a reduction in the risk of intestinal and mammary cancers [3]. The consumption of eastern varieties of seaweed is becoming increasingly popular in western countries, including the United Kingdom, where oriental dishes are growing in popularity. Algae can accumulate and biotransform inorganic arsenic to organic arsenic, and organic arsenic is the most common form of arsenic in algae, where it occurs predominantly as arsenosugars [4, 5]. Inorganic arsenic concentrations in algae seldom exceed 1 mg/kg, with the exception of the edible brown seaweed* Hizikia fusiformis *which contains high levels of inorganic arsenic [6]. On the basis of this information, some committees from different countries such the Canadian Food Inspection Agency in 2001, the UK Food Standards Agency in 2004 and 2010, the European Commission in 2004, the Food Standards Australia-New Zealand in 2004, and the Hong Kong Centre for Food Safety in 2005 have issued the warning and advised the public not to eat* hijiki *[7]. In 2004, the Japanese Ministry of Health, Labour and Welfare advised consumers not to eat “too much”* hijiki*. However, at the same time, the Japanese Ministry also identified* hijiki *as a good source of dietary fiber and essential minerals [7]. And, to date, there have been no incidents of inorganic arsenic poisoning resulting from the consumption of* hijiki* or any other species of seaweeds.

So the levels of the different arsenic species should ideally be determined* in vivo*. Human* in vivo* studies, however, are costly, time-consuming, and complicated to perform, and the results of these studies can vary considerably. Laboratory animal* in vivo* studies are less expensive but limited by the uncertainties associated with the differences between the metabolisms of animals and man. As an alternative to human and animal* in vivo* studies, the bioavailabilities of trace metals have also been estimated using simple, rapid, and inexpensive* in vitro* enzymatic methods [8–12]. It is necessary to understand exactly what species of arsenic are present during the digestion processes in the stomach and the intestine.

Detailed reviews of the analytical methods available for arsenic speciation can be found in the literature [1, 2]. The technique most commonly used in arsenic speciation analysis involves the separation of the different arsenic species by high-performance liquid chromatography (HPLC), followed by detection of the separated species by inductively coupled plasma mass spectrometry (ICP-MS) [13, 14].

The aim of present study was to analyze the levels of the four main toxic arsenic species As (III), As (V), MMA, and DMA in five different types of edible marine algae from China using* in vitro* digestion system determined by HPLC-ICP-MS. The five edible seaweeds used in the current study where the brown algae* Laminaria japonica*, red algae* Porphyra yezoensis*, brown algae* Undaria pinnatifida*, brown algae* H. fusiformis*, and green algae* Enteromorpha prolifera*. It was envisaged that the results of this study would provide crucial information for the risk assessment of arsenic species in edible seaweeds.

## 2. Materials and Methods

### 2.1. Samples

*L. japonica*,* P. yezoensis*,* U. pinnatifida*,* H. fusiformis, *and* E. prolifera* were randomly purchased from the supermarket during August and October 2012 in Qingdao, Shandong Province, China. All five samples were oven-dried at 60°C to constant weight and then carefully grounded and homogenized in the laboratory prior to their analysis.

### 2.2. Reagents and Standards

All of the solutions were prepared with doubly deionized water, which was obtained from a Millipore water purification system (18.2 MΩ cm^−1^ resistivity). Concentrated nitric acid (69-70%) and hydrogen peroxide (31%) were purchased from Beijing Chemical Reagent Company (Beijing, China) and used for total arsenic digestion of the seaweed samples. NaH_2_PO_4_, CH_3_COONa, EDTA-2Na, and KNO_3_ were purchased from Sigma (St. Louis, MO, USA) and used for the analysis of arsenic species.

Commercial standard solutions of arsenite [As (III)] (GBW08666), arsenate [As (V)] (GBW08667), monomethylarsonic acid (MMA) (GBW08668), and dimethylarsinic acid (DMA) (GBW08669) were purchased from the National Standard Material Management Committee of China. All of the stock solutions were held at 4°C, and further diluted solutions were prepared for analysis on a daily basis. A standard arsenic solution with a certified arsenic concentration of 1000 mg/L was supplied by the National Standard Material Management Committee and used to calibrate the ICP-MS equipment for the determination of the total arsenic content.

The biological chemicals, including uric acid, mucin, albumin bovine, pepsin, pancreatin, lipase, and bile, were all purchased from Sigma. According to the literature [11, 12, 15], the compositions and amounts of the inorganic and organic constituents, including the digestive enzymes, were designed on the basis of human physiology. The detailed components were listed in Table 1. pH values of all the solutions were adjusted by HCl or NaOH, and the total volume of the digest solution was diluted to 500 mL with ultrapure water. The biomimetic digestion solutions were stored at 4°C before use.

To avoid metal contamination, all of the glassware used in the current study was washed and kept for at least 24 h in 15% (v/v) nitric acid before being rinsed at least three times with deionized water prior to their use.

### 2.3. Instruments

The seaweed samples were digested using a microwave-assisted digestion system (CEM Mars 40, USA) and the total arsenic levels were determined using an inductively coupled plasma mass spectrometer (ICP-MS) (PerkinElmer, ELAN DRC II, USA). The arsenic species were analyzed by HPLC using a Series 200 System (PerkinElmer) coupled to an ICP-MS system. The pH values were measured using a Mettler Toledo 320-S pH-meter (Mettler Toledo Co., China). A temperature-consistent oscillating water bath (SHA-B, GuoHua Co., China) and a centrifuge (Eppendorf 5810R, Germany) were used.

### 2.4. Total Arsenic Determination

Samples (0.5 g dry weight) were quantitatively digested in quartz high pressure closed vessels using concentrated nitric acid (5 mL) and hydrogen peroxide (2 mL) and a microwave-assisted digestion system. The digested liquors were quantitatively transferred to graduated test tubes and made up to 50 mL with deionized water. The total arsenic levels were measured using ICP-MS. Three replicates were performed for each species of seaweed. At least two different blanks were performed for each set of microwave conditions.

Quality assurance and quality control (QA/QC) for total arsenic content in seaweeds were conducted using two certified reference materials, including GBW 08517 Kelp and GBW 08521 Laver, which were purchased from the National Standard Material Management Committee of China.  Table 3 shows the analytical results (mean ± SD) for five independent analyses of two certified reference materials (CRM). It is seen that the results agreed with the certified values.

### 2.5. Arsenic Speciation Analysis in Seaweed

The dried and homogenized seaweed samples (0.5 g) were accurately weighed in triplicate into 50 mL (polypropylene) tubes followed by 38 mL of deionized water and extracted using ultrasonic irradiation over a period of 40 min. To remove the protein, 2 mL of 3% (v/v) CH_3_COOH was added and the resulting mixture was mixed completely. The samples were then held at 4°C for 5 min before being centrifuged for 10 min (8000 ×g, 4°C). The supernatants were then collected and filtered through a 0.45 *μ*m membrane before being analyzed by HPLC-ICP-MS. At the same time, at least two different blanks were performed for each set of conditions.

### 2.6. Experimental Design of* In Vitro* Bionic Digestion Procedure

A method of “*in vitro *digestion” has been used in the current study to analyze the different species of arsenic present in several edible marine algae from China. Briefly, the marine algae were digested in a simulated digestive system, where the conditions were consistent with those found in the stomach or intestine, including the body temperature, acidity of the stomach or intestine, and the presence of inorganic and organic materials (digestive enzymes included for whole-bionic digestion) normally found in the stomach or intestine. Active stirring was used to simulate the movements inside the stomach and intestine. Furthermore, the stirring time was controlled based on the time that edible marine algae would typically require for its passage through the stomach and intestine of a healthy human. Following the* in vitro* digestion process, the arsenic species in the digestive juices of the stomach and intestine were analyzed.

**Figure 1:**
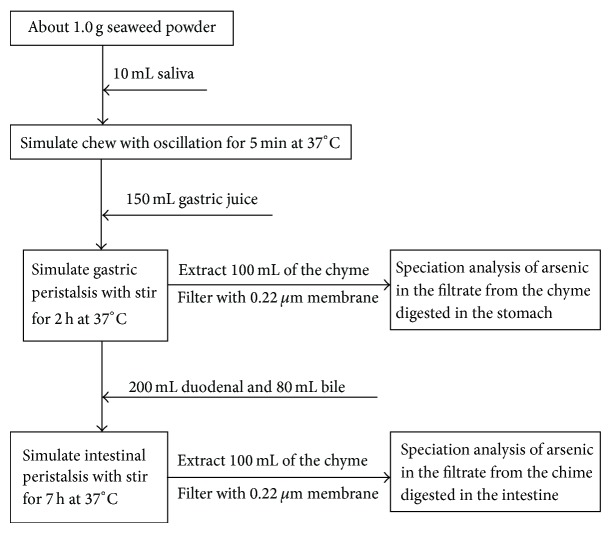
Schematic diagram of the* in vitro* bionic digestion of the seaweed powder.

The* in vitro* bionic digestion procedure was performed, in triplicate, by weighing 1.0 g powdered seaweed into 500 mL Erlenmeyer flasks. The gastrointestinal tract was simulated for the mouth, stomach, and small intestine. The seaweed samples were digested at 37°C under the action of acid and digestive enzymes of the stomach and intestine. Gastric and intestinal peristalses were simulated by stirring for 5 min, 2 h, and 7 h, respectively, according to the times the food remains in the mouth, stomach and intestine. A schematic representation of the* in vitro* digestion model is presented in Figure 1 according to Li et al. [11].

### 2.7. Arsenic Species Analysis by HPLC-ICP-MS

**Figure 2:**
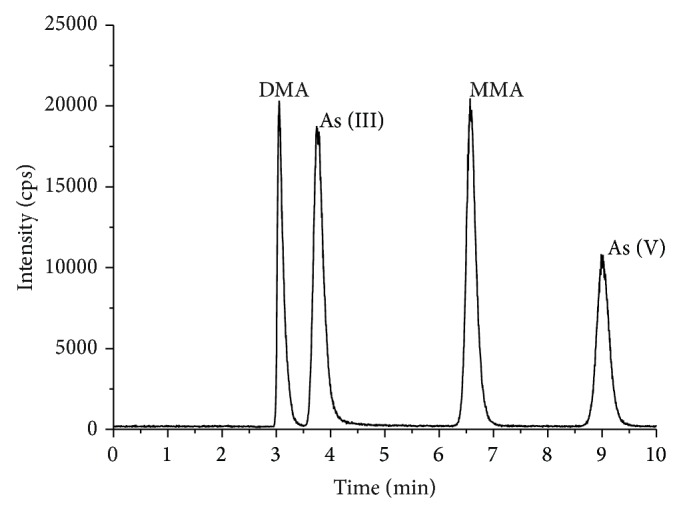
Separation of the four arsenic species As (III), As (V), MMA, and DMA using HPLC coupled online to ICP-MS.

Anion-exchange HPLC conditions were used to obtain the separation of four arsenic species (As (III), As (V), MMA, and DMA) in a single chromatographic run. The operating conditions of the ICP-MS and the chromatographic systems are summarized in Table 2. The arsenic compounds in the extracts were identified by matching their retention times with those of the standards. It is noteworthy that the concentrations of arsenic species reported in the current study were not based on all of the arsenic compounds but only on the ^75^As species. ICP-MS measurements are not supposed to be influenced by the type of arsenic compound. The concentrations of all of the arsenic species in the current study were compared on the basis of ^75^As. All species were eluted within 10 min. The retention times for arsenic species were 3.07 min for DMA, 3.76 min for As (III), 6.60 min for MMA, and 9.00 min for As (V). A typical chromatogram obtained using those conditions is shown in Figure 2.

**Figure 3:**
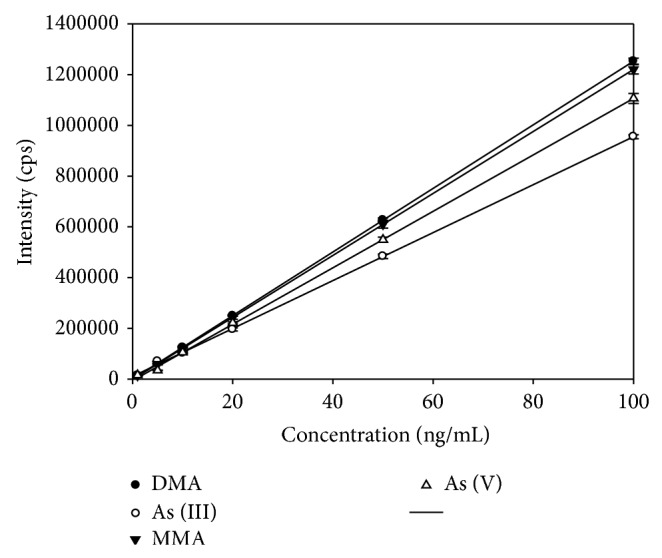
Calibration curves obtained for the arsenic species As (III), As (V), MMA, and DMA with HPLC-ICP-MS (mean of three replicates).

Calibration curves for the four arsenic species were established for the concentration range of 1 to 100 ng/mL. The levels of concentration were 1, 5, 10, 20, 50, and 100 ng/mL as arsenic. Peak area was used for quantification. The calibration curves are shown in Figure 3. A linearity study of the calibration curves was performed for each compound. Correlation coefficients were higher than 0.998 for all species and the relative standard deviations for the points of the calibration graphs were between 3.2% and 7.8% for 1 and 5 ng/mL and between 0.8% and 4.5% from 10 to 100 ng/mL. The repeatability of the method was also studied for each compound at six concentration levels. Three replicates of the calibration were performed with three injections for every standard. The relative standard deviation did not exceed 7.8%.

### 2.8. Statistical Analysis

Data were expressed as means and standard deviations. The data were statistically analyzed with one-way analysis of variance using the SPSS statistics software package (Version 16.0). Least significant difference (LSD) was performed at a 95% confidence level.

## 3. Results and Discussion

### 3.1. Total Arsenic in Seaweeds

The total arsenic concentrations of the seaweed samples were determined by ICP-MS and the total arsenic values in* L. japonica*,* P. yezoensis*,* U. pinnatifida*,* H. fusiformis*, and* E. prolifera* were shown in Table 3. The brown seaweed* H. fusiformis *had highest total As content, and the green seaweed* E. prolifera* had lowest total As level. Thus, the brown seaweeds contained significantly higher concentrations of arsenic than the green seaweeds, and these results were in accordance with those previously published in the literature [16, 17]. The different levels of arsenic in the algae were attributed to the arsenic absorption, retention, and excretion capacities associated with the different species [18]. The most abundant polysaccharide found within the living cells of brown algae is alginate, which accounts for 10–70% of the dry weight of the cells. Alginic acid can absorb metals and metalloids in the marine environment to a greater or lesser extent [19].

#### 3.1.1. Arsenic Speciation Analysis in the Water Extracts

A variety of different extraction solvents have been used to extract arsenic compounds from biological materials, with methanol and water being the most commonly used solvents. Given that the arsenic compounds investigated in the current study are very polar, water was used as the extraction solvent to provide the best general extraction conditions [1]. The results are shown in Table 4. As (III) was found in* L. japonica* and* U. pinnatifida* in concentrations of 0.20 and 0.10 mg/kg, respectively. The concentrations of As (V) in* H. fusiformis *and* E. prolifera* were 23.0 mg/kg and 0.15 mg/kg, respectively. DMA was found in all of the samples, with the highest concentration found in* L. japonica*, followed by* P. yezoensis*. The lowest concentration of DMA was found in* E. prolifera*. MMA was only found in* H. fusiformis*, where its concentration was very low (0.10 mg/kg).

The results revealed that DMA was present in the water extracts of all five of the seaweeds tested, with the water samples providing an accurate representation of the arsenic species actually present in the five samples. As (V) existed in* H. fusiformis *and* E. prolifera*, whereas As (III) was found in* L. japonica* and* U. pinnatifida*. Similar to previous studies [6],* hijiki* seaweed (*H. fusiformis*) had high content of As (V) which occupied about 27.9% of the total As in the present study. Algae have been reported to take up and bioaccumulate As (V) from seawater. As (V) can then be reduced to As (III) and can be detoxified following a process that ultimately leads to reduction, oxidative methylation, and adenosylation processes and the formation of methylarsonate, dimethylarsinate, and arsenosugars, respectively [20]. Studies carried out in the brown algae* Fucus serratus* [20] and* Chlorella vulgaris* [21] have provided good support for the existence of this pathway. Arsenic therefore exists in most species of algae, where it can be converted to dimethylarsinate and arsenosugars. This is also true for the seaweeds* L. japonica*,* P. yezoensis*,* U. pinnatifida*, and* E. prolifera.* In contrast, however, the main inorganic arsenic species found in* H. fusiformis* was As (V). In this particular case, the* H. fusiformis *presumably absorbs the As (V) and holds it in this form without subjecting it to the reduction and oxidative methylation processes that are regarded as detoxification strategies for seaweed [22]. On the basis of the great toxicity of As (V), the subcellular distribution pattern of As (V) in* hijiki* may possess special characteristics that may help to explain the high capacity of this species for the inorganic arsenic, because many studies have reported that the subcellular distribution and chemical form of heavy metals may be associated with metal tolerance and detoxification in plants [23]. Further studies towards developing a better understanding of the subcellular distribution of As (V) in* hijiki* are therefore required.

The current results demonstrate that the levels of the inorganic arsenic species present in the seaweeds were much lower than the limitations imposed by the regulatory authorities in China and several other countries [6], except for* H. fusiformis*, where inorganic arsenic-As (V) was identified as the main arsenic species present in the deionized water extracts. According to Rose et al. [24], consumption of a 25 g* hijiki* seaweed would therefore increase the intake of inorganic arsenic by around 30 times. In fact, although a case of suspected arsenic poisoning due to an herbal kelp supplement has been reported in the USA [7], seaweeds including* hijiki* remains a popular food in some Asian countries, especially in Japan, and no accidents have been proved to be related with the high concentration of inorganic arsenic.

### 3.2. Arsenic Speciation Analysis in* In Vitro* Gastrointestinal Digestive Juice

Arsenic's contradictory status as poisonous or safe is well known and is based on its toxicity being dependent on two factors, namely, (1) its chemical form, or speciation, and (2) its absorption into a living organism, or bioavailability. To calculate the risks associated with arsenic ingested in seaweed, it is necessary to identify the nature and quantity of the arsenic species present in the seaweed and the metabolites formed as a consequence of the expected metabolic activities. It is therefore necessary to analyze arsenic speciation in simulated gastrointestinal media.

**Figure 4:**
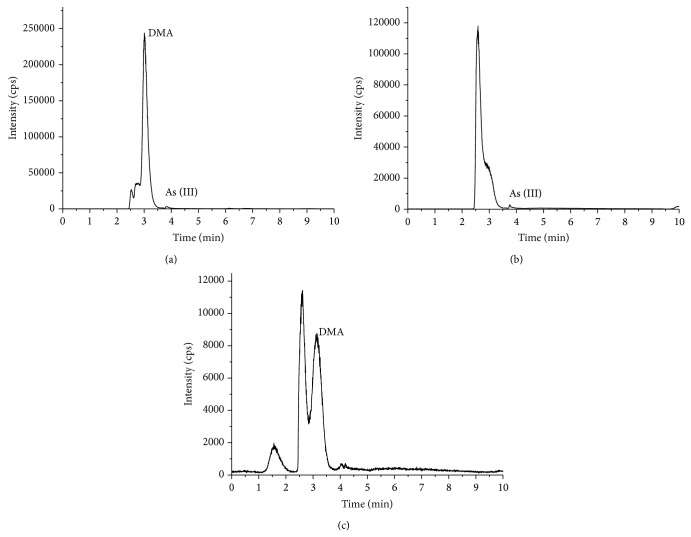
Arsenic speciation analysis for* L. japonica* ((a) the deionized water extracts, (b) the gastric extracts, and (c) the intestinal extracts).

DMA was determined to be the main arsenic species in the deionized water extracts of* L. japonica* (Figure 4). About 0.19 mg/kg of As (III) was extracted from the gastric extracts. The DMA concentration in the intestinal extracts, based on ^75^As, was found to be 0.64 mg/kg. The details of these analyses are shown in Table 4.

**Figure 5:**
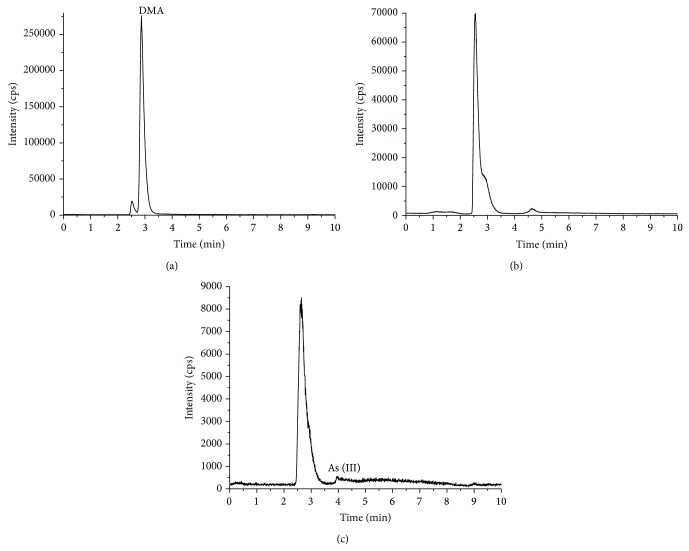
Arsenic speciation analysis for* P. yezoensis* ((a) was the deionized water extracts; (b) was the gastric extracts; (c) was the intestinal extracts).

DMA was also determined to be the main arsenic species in the deionized water extracts from* P. yezoensis* (Figure 5). None of the four arsenic species were found in the gastric extracts, whereas 0.31 mg/kg of As (III) was extracted from the intestinal extracts (see Table 4).

**Figure 6:**
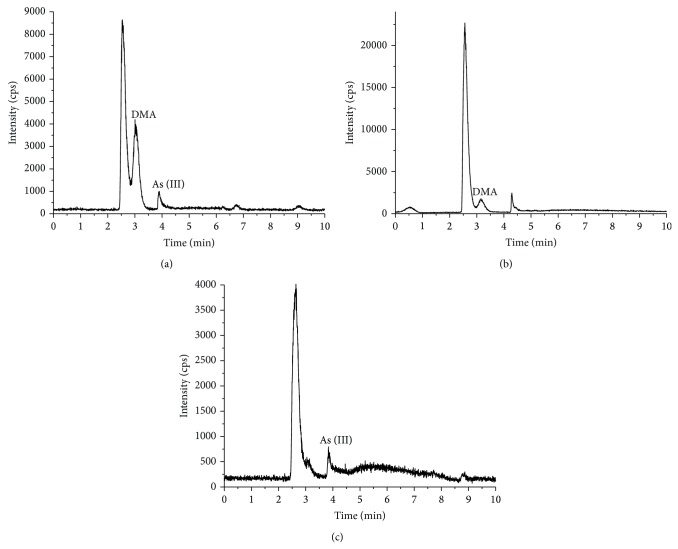
Arsenic speciation analysis for* U. pinnatifida* ((a) was the deionized water extracts; (b) was the gastric extracts; (c) was the intestinal extracts).

The arsenic compounds found in* U. pinnatifida* are shown in Figure 6. DMA was detected in both the water and the gastric extracts at concentrations of 0.74 and 0.44 mg/kg, respectively. The level of As (III) in the intestinal extracts was found to be 0.10 mg/kg (see Table 4).

**Figure 7:**
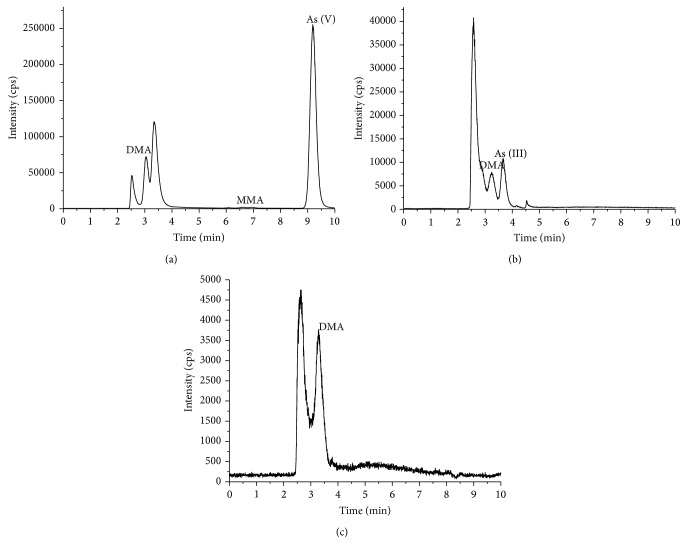
Arsenic speciation analysis for* H. fusiformis *((a) the deionized water extracts, (b) the gastric extracts, and (c) the intestinal extracts).

The arsenic components of* H. fusiformis *were found to be much more complicated and varying (Figure 7). In contrast to the other seaweeds, the major arsenic species found in the deionized water from* H. fusiformis *was As (V), with only a low concentration of MMA being detected (Table 4). Interestingly, however, As (V) was not detected in the gastric or intestinal extracts. Instead, DMA and As (III) were extracted from the gastric digestion process after 2 h in levels of 1.68 and 1.47 mg/kg, respectively. Following the intestinal digestion, however, only 2.22 mg/kg of DMA could be extracted.

**Figure 8:**
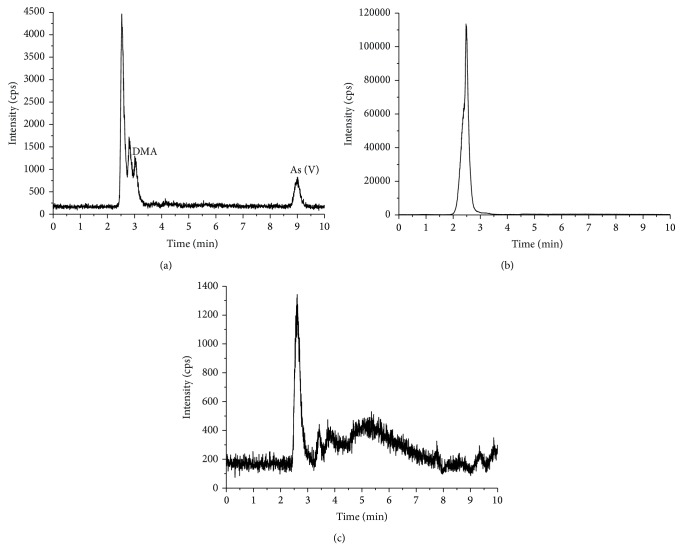
Arsenic speciation analysis for* E. prolifera *((a) the deionized water extracts, (b) the gastric extracts, and (c) the intestinal extracts).

The levels of DMA and As (V) detected in the deionized water extracts of* E. prolifera* were found to be 0.16 and 0.15 mg/kg, respectively, based on ^75^As. None of the four arsenic species of interest were detected in the gastric or intestinal extracts from* E. prolifera* (Figure 8 and Table 4).

The arsenic species in the seaweed after being digested by stomach or intestine have not been researched by now and in fact, this is more important for the risk assessment of the seaweed which is regarded as favorite food. On the basis of the results from the current study, it can be concluded that different arsenic species were present in the water and biomimetic digestion extracts. The greatest and most interesting difference was that the gastric and intestinal extracts from the five seaweeds did not contain any As (V) or MMA, including the brown seaweed* H. fusiformis *which has been proved to contain high content of As (V). In another words, As (V) could not be released from* H. fusiformis *after being digested by stomach or intestine. However, we found that the gastric extracts of* H. fusiformis *contained 1.47 mg/kg of As (III), while not any inorganic arsenic was extracted after the intestinal digestion. In addition, the present study proved that there is no As (III) in the water extracts of* H. fusiformis *which may provide an accurate representation of the arsenic species in samples. These differences may be attributed to the As (V) being reduced to As (III) in the acidic gastric extracts.

Furthermore, the current results revealed that As (III) was found in the water extracts of* L. japonica* and* U. pinnatifida*, and, after the presented biomimetic digestion process, all of As (III) content was fully extracted since the concentration in the water extracts from both seaweeds was similar to those of the biomimetic digestion extracts. A surprising result is that As (III) could also be detected in the intestinal extracts of* P. yezoensis* which had not any inorganic arsenic in the water extracts. For the* E. prolifera*, however, none of the four arsenic species could be detected following the biomimetic digestion process, although about 0.16 mg/kg DMA and 0.15 mg/kg As (V) were determined in the water extracts. The current results successfully demonstrate that there are significant differences in the types of arsenic species present in the deionized water and gastrointestinal extracts of the five seaweeds studied. These differences may depend on different seaweed species and arsenic species. Besides the action of the acidic gastric juices and alkalescent intestinal juices, the gastrointestinal enzymes play a significant activity in digestion of mineral complexes [25]. Some of the DMA could also be extracted by the biomimetic digestion process, but their contents were much lower than those in the water extracts.

### 3.3. Risk Assessment of Four Arsenic Species in Edible Seaweeds Based on* In Vitro* Studies

The risk assessment of the inorganic arsenic based on the* in vitro* results may be more accurate when considering present results that the significant differences existed in the species of arsenic and the content of different arsenic species in the deionized water and gastrointestinal extracts of five seaweeds. According to the seventy-second report of the Joint FAO/WHO Expert Committee on Food Additives published by the World Health Organization 2011 [26], the dietary inorganic arsenic PTWI (provisional tolerable weekly intake) of 15 *μ*g/kg bw (2.1 *μ*g/kg bw per day) confirmed at its thirty-third meeting (1988) was withdrawn, because it is in the region of the BMDL_0.5_ (lower 95% confidence limit for the benchmark dose for 0.5% increased incidence of cancer over background) which was computed to be 3.0 *μ*g/kg bw per day (2.0–7.0 *μ*g/kg bw per day based on the range of estimated total dietary exposure). If an adult (60 kg bw) consumed the maximum suggested amount of* hijiki* seaweed (25 g) one day with the maximum concentration of inorganic arsenic (1.47 mg/kg) after the gastrointestinal digestion, it would lead to an inorganic arsenic intake of 0.6 *μ*g/kg bw per day which is much lower than the BMDL_0.5_. On the other hand, arsenic may serve an essential role in growth and nutrition. Based on studies of arsenic deprivation in laboratory animals, an arsenic requirement for humans eating 2000 kcal/day has been estimated to be in the range of 12 to 25 *μ*g/day. Deficiencies related to low arsenic intakes would most likely appear in individuals with altered arsenic homeostasis or metabolic stress [27]. However, chronic daily inorganic arsenic intakes of 600 *μ*g or more have been associated with adverse effects [28]. So it could be included that the inorganic arsenic in the seaweeds including* hijiki* seaweed brought little health hazards. However, it still needs further research to provide more related information. Yokoi and Konomi found that feeding rats a 3%* hijiki* (30 g powder) diet lasting for 7 weeks led to a marked accumulation of arsenic in blood and tissues and evoked a high body temperature and abnormal blood biochemistry [7]. We speculated that 30 g* hijiki* powder diet lasting for 7 weeks was overloaded for the rats; so under the above experimental conditions, the inorganic arsenic released by digestion have exceeded the safety limitations for the rats which must be different from the human's; as a result, the high As (III) may be one reason for the harmful effects on the experimental rats. For DMA and MMA, there are no limitations in any countries by now. In addition, the toxicity of MMA or DMA is much lower than the inorganic arsenic (the latter is about 100 times greater than the former) [2]; so based on the toxicity ratio of DMA to inorganic arsenic, the content of DMA after gastrointestinal digestion is so low as not to induce damage to human health.

## Figures and Tables

**Table 1:** Components of saliva, gastric juice, duodenal, and bile.

	Saliva	Gastric juice	Duodenal	Bile
Inorganic materials	10 mL 89.6 g/L KCl	15.7 mL NaCl 175.3 g/L	40 mL NaCl 175.3 g/L	30 mL NaCl 175.3 g/L
	10 mL 20 g/L KSCN	3.0 mL NaH_2_PO_4_ 88.8 g/L	40 mL NaHCO_3_ 84.7 g/L	68.3 mL NaHCO_3_ 4.7 g/L
	10 mL 88.8 g/L NaH_2_PO_4 _	9.2 mL KCl 89.6 g/L	10 mL KH_2_PO_4_ 8 g/L	4.2 mL KCl 89.6 g/L
	10 mL Na_3_PO_4_ 57 g/L	18 mL CaCl_2_·2H_2_O 22.2 g/L	6.3 mL KCl 89.6 g/L	0.2 mL HCl 37% g/g
	1.7 mL NaCl 175.3 g/L	10 mL NH_4_Cl 30.6 g/L	10 mL MgCl_2_5 g/L	10 mL CaCl_2_·2H_2_O 22.2 g/L
	1.8 mL NaOH 40 g/L	8.3 mL HCl 37% (g/g)	0.18 mL HCl 37% (g/g)	
			9 mL CaCl_2_·2H_2_O 22.2 g/L	

Organic materials	8 mL urea 25 g/L	10 mL glucose 65 g/L	4 mL urea 25 g/L	
		10 mL glucuronic acid 2 g/L		
		3.4 mL urea 25 g/L		
		10 mL 33 g/L glucosamine hydrochloride		

Bioenzymes	145 mg *a*-amylase	1 g bovine serum albumin	1 g bovine serum albumin	1.8 g bovine serum albumin
	15 mg uric acid	1 g pepsin	3 g pancreatin	6 g bile
	50 mg mucin	3 g mucin	0.5 g lipase	

pH	6.5 ± 0.2	1.07 ± 0.07	7.8 ± 0.2	8.0 ± 0.2

**Table 2:** Operating conditions of the ICP-MS and the chromatographic systems.

Inductively coupled plasma mass spectrometry (ICP-MS)	
Radio frequency power	1300 W
Sampler and skimmer cones	Nickel
Argon flow rates:	
Plasma	15 L/min
Auxiliary	0.86 L/min
Nebulizer	0.96 L/min
Date acquisition mode	Graphics (signal intensity versus time)
Analytical mass (amu)	^ 75^As
Chromatography	
Anion exchange	
Guard column	IonPac AG 19 (50 × 4 mm)
Analytical column	IonPac AS 19 (250 × 4 mm)
Mobile phase	10 mmol/L CH_3_COONa, 3 mmol/L KNO_3_, 2 mmol/L NaH_2_PO_4_, and 0.2 mmol/L EDTA-2Na adjusted to pH 10.7 with 4% NaOH
Flow rate	1.0 mL min^−1^
Injection volume	30 *μ*L

**Table 3:** Total arsenic contents of the five seaweeds and two certificated reference materials (mg/kg, dry weight).

Seaweed sample	Total As (*n* = 3)	CRM	Total As (*n* = 5)	Certified values
*L. japonica *	63.7 ± 4.1^a^	GBW 08517	13.2 ± 1.8	13.9 ± 2.4
*P. yezoensis *	28.1 ± 2.8^b^	GBW 08521	40.2 ± 2.5	41 ± 3
*U. pinnatifida *	30.1 ± 2.2^b^			
*H. fusiformis *	82.5 ± 3.6^c^			
*E. prolifera *	6.87 ± 0.48^d^			

Note: different letters mean significant difference at *P* < 0.05 level.

**Table 4:** Four arsenic species contents of the water extracts and gastrointestinal extracts from the five seaweeds (calculations based on the As contents, mg/kg, dry weight) (*n* = 3).

	DMA	As (III)	MMA	As (V)
*L. japonica *				
Water extracts	36.7 ± 3.2	0.20 ± 0.03	0.0 ± 0.0	0.0 ± 0.0
Gastric extracts	0.0 ± 0.0	0.19 ± 0.02	0.0 ± 0.0	0.0 ± 0.0
Intestinal extracts	0.64 ± 0.04	0.0 ± 0.0	0.0 ± 0.0	0.0 ± 0.0

*P. yezoensis *				
Water extracts	19.1 ± 2.0	0.0 ± 0.0	0.0 ± 0.0	0.0 ± 0.0
Gastric extracts	0.0 ± 0.0	0.0 ± 0.0	0.0 ± 0.0	0.0 ± 0.0
Intestinal extracts	0.0 ± 0.0	0.31 ± 0.05	0.0 ± 0.0	0.0 ± 0.0

*U. pinnatifida *				
Water extracts	0.74 ± 0.02	0.10 ± 0.01	0.0 ± 0.0	0.0 ± 0.0
Gastric extracts	0.44 ± 0.04	0.0 ± 0.0	0.0 ± 0.0	0.0 ± 0.0
Intestinal extracts	0.0 ± 0.0	0.10 ± 0.01	0.0 ± 0.0	0.0 ± 0.0

*H. fusiformis *				
Water extracts	4.38 ± 0.26	0.0 ± 0.0	0.10 ± 0.02	23.0 ± 2.5
Gastric extracts	1.68 ± 0.15	1.47 ± 0.20	0.0 ± 0.0	0.0 ± 0.0
Intestinal extracts	2.22 ± 0.18	0.0 ± 0.0	0.0 ± 0.0	0.0 ± 0.0

*E. prolifera *				
Water extracts	0.16 ± 0.02	0.0 ± 0.0	0.0 ± 0.0	0.15 ± 0.02
Gastric extracts	0.0 ± 0.0	0.0 ± 0.0	0.0 ± 0.0	0.0 ± 0.0
Intestinal extracts	0.0 ± 0.0	0.0 ± 0.0	0.0 ± 0.0	0.0 ± 0.0
